# Internalized homophobia, mental health, sexual behaviors, and outness of gay/bisexual men from Southwest China

**DOI:** 10.1186/s12939-017-0530-1

**Published:** 2017-02-17

**Authors:** Wenjian Xu, Lijun Zheng, Yin Xu, Yong Zheng

**Affiliations:** 1 Key Laboratory of Cognition and Personality (MOE), Southwest University, Chongqing, China; 2 Department of Psychology, King’s College London, London, UK; 3 Faculty of Psychology, Southwest University, Chongqing, 400715 China

**Keywords:** Internalized homophobia, Mental health, Sexual behaviors, Outness, Chinese gay/bisexual men

## Abstract

**Background:**

Social attitudes toward male homosexuality in China so far are still not optimistic. Sexual minorities in China have reported high levels of internalized homophobia.

**Methods:**

This Internet-based study examined the associations among internalized homophobia, mental health, sexual behaviors, and outness among 435 gay/bisexual men in Southwest China from 2014 to 2015. Latent profile analysis, confirmatory factor analysis, univariate logistic regression, and separate multivariate logistic regression analyses were conducted.

**Results:**

This descriptive study found the Internalized Homophobia Scale to be suitable for use in China. The sample demonstrated a high prevalence of internalized homophobia. Latent profile analysis suggested a 2-class solution as optimal, and a high level of internalized homophobia was significantly associated with greater psychological distress (Wald = 6.49, AOR = 1.66), transactional sex during the previous 6 months (Wald = 5.23, AOR = 2.77), more sexual compulsions (Wald = 14.05, AOR = 2.12), and the concealment of sexual identity from others (Wald = 30.70, AOR = 0.30) and parents (Wald = 6.72, AOR = 0.49).

**Conclusions:**

These findings contribute to our understanding of internalized homophobia in China, and highlight the need to decrease gay-related psychological stress/distress and improve public health services.

**Electronic supplementary material:**

The online version of this article (doi:10.1186/s12939-017-0530-1) contains supplementary material, which is available to authorized users.

## Background

Internalized homophobia is defined as the self-hatred and shame of homosexually oriented individuals that has been incorporated into their belief system [1]; it is also a source of stress. The construct includes negative global attitudes toward homosexuality, discomfort with disclosure of sexual orientation to others, disconnectedness from other lesbian, gay, and bisexual (LGB) individuals, and discomfort with same-sex sexual activity [1, 2]. Accordingly, Meyer has developed the Internalized Homophobia Scale, which has been widely used among sexual minorities in Western countries [3–8].

Social attitudes toward male homosexuals in Western society have improved considerably in the past three decades [9]. The acceptance of legalized same-sex marriage, which almost eliminated partnership differences between homosexual and heterosexual couples in the United States [10, 11] and other developed countries [12, 13], has been accompanied by a decrease in homophobic behavior and internalized homophobia. For example, the number of gay/straight alliances in United States secondary schools rose from 100 in 1995 to more than 3,000 in 2007, and most high school seniors graduating in 2006 favored legalizing same-sex marriage or civil unions [2].

Social attitudes toward male homosexuality in China so far are still not optimistic. First, Chinese traditional values emphasize the continuity of bloodlines. Second, HIV infection has increased at an alarming rate among gay males in recent years [14]. In this context, some Chinese people tend to associate gay men with AIDS [15]. Third, same-sex marriage is illegal and sexual minorities still experience stigma, prejudice, and the occurrence of negative events due to their sexual orientation [16]. Even same-sex behaviors may lead to rejection by one’s family and problems in the work setting [17]. As a result, some gay men choose to conceal their same-sex orientation (especially from their parents) [16] and choose opposite-sex marriage to ease the stress of being members of this minority (i.e., internalized homophobia). A study comparing men who have sex with men (MSM) from outside China with MSM in China using a global online survey found that MSM in China reported higher levels of internalized homophobia [18].

Little empirical information is available about the associations between internalized homophobia, mental health, sexual behavior, and substance use among Chinese gay and bisexual individuals. Previous studies of Chinese MSM have indicated that internalized homophobia was a major barrier to accessing HIV prevention and care services, and it was negatively associated with being tested for HIV [18]. However, that study did not examine the associations between internalized homophobia and mental health. Research studies conducted in developed countries have found that higher levels of internalized homophobia were associated with higher levels of mental health problems (i.e., depression and anxiety) [2, 19]. Additionally, internalized homophobia was found to be associated with practicing HIV at-risk sexual behaviors [1, 20], such as condomless sex, multiple sex partners, and unprotected anal sex among male homosexuals. In addition, previous reviews of studies have reported inconsistent conclusions regarding the relationship between internalized homophobia and substance/alcohol use. Some studies have found an association between higher levels of internalized homophobia and increased alcohol use [21], and substance use [21], such as barbiturates and amphetamines, among male individuals, whereas other studies have reported conflicting findings [22, 23].

Overall, there are differences in cultural and social contexts between Western and Chinese male sexual minorities, especially with respect to the norms and social meanings regarding one’s number of partners (i.e., multiple sex partnerships), which have the potential to affect the expression of individuals’ internalized homophobia. Understanding the issue of internalized homophobia should improve interventions that raise levels of mental health and public health (i.e., HIV risks) among gay and bisexual men. To our current knowledge, two published studies have addressed internalized homophobia among Chinese gay and bisexual men [18, 24], both of which were focused on the associations between internalized homophobia and their rates of HIV testing. However, neither of these studies provided a detailed analysis of internalized homophobia as a risk factor for psychological distress and engaging in unprotected sex among Chinese male sexual minorities.

Previous studies have examined internalized homophobia as a correlate of mental health and health risks in developed countries [3, 5, 7, 21]. Among MSM in China, there has been a dramatic increase in new HIV infections, especially among MSM in Southwest China [14]. Furthermore, MSM could potentially act as a bridge to transmit HIV to the general female population through unprotected sex with females because of their inconsistent condom use [25]. In addition, internalized homophobia has been found to be associated with sexual compulsivity [20, 26]. Thus, we first examined the suitability of the Internalized Homophobia Scale. Second, we examined the associations among internalized homophobia, psychological distress, sexual behaviors, and outness. We hypothesized that internalized homophobia would be positively associated with psychological distress, concealment of sexual orientation, sexual compulsivity, and unprotected sex among Chinese gay and bisexual men. The findings of the study will contribute to a better understanding of internalized homophobia in China, and highlight the need to decrease gay-related psychological stress/distress and improve public health services.

## Methods

### Design and participants

The study was designed to determine the associations between internalized homophobia, mental health, sexual behaviors, outness, and substance use among the gay and bisexual male population in Southwest China. A cross-sectional survey was conducted online in 2014 and 2015. Participants were recruited over a period of three months using advertisements on gay chat-room websites, Blued and Zank application software (two popular gay social software applications in China), and QQ groups. Participants completed an Internet-based survey questionnaire via the professional survey website, Wenjuan xing. The initial dataset consisted of 513 respondents.

Participants were eligible for the study if they were male, age 18 years or older, gay or bisexual, and residents of one of the recruitment regions in Southwest China. The website recorded each user’s city address, and the addresses of the respondents living in Sichuan, Chongqing, Yunnan, and Guizhou Provinces were included. A total of 435 participants were eligible for this study.

### Measures

#### Socio-demographic data

Basic socio-demographic information, including age, highest educational level attained, occupation, monthly salary, and current relationship status, was collected.

#### Internalized homophobia

Internalized homophobia was measured using the 9-item Internalized Homophobia Scale that assesses the extent to which gay/bisexual individuals reject their sexual orientation, uneasiness about their same-sex desires, and seeking to avoid same-sex personal and social involvement and sexual feelings [1, 6, 19, 27]. Sample items include “I have tried to stop being attracted to men in general” and “I would like to get professional help in order to change my sexual orientation from gay/bisexual to straight.” Responses are made on a 5-point Likert scale ranging from 1 (*strongly disagree*) to 5 (*strongly agree*). Scores range from 9 to 45, with higher scores indicating higher levels of internalized homophobia. The Chinese version of the scale was translated by one English professional translator and two psychology graduate students independently, and then consensus was reached. Previous research has indicated that the scale has acceptable internal consistency reliability [1, 27]. Cronbach’s α for the Chinese version of the scale in the present study was 0.88.

#### Mental health

The 6-item Kessler Psychological Distress Scale (K6) was used to measure respondents’ non-specific psychological distress during the past month [28]. The items measure depression and anxiety symptoms. Examples of items are “How often did you feel worthless?” and “How often did you feel anxious?” Responses are made using a 5-point Likert-type scale ranging from 1 (*none of the time*) to 5 (*all of the time*). Scores range from 6 to 30, with higher scores indicating higher levels of psychological distress. The internal consistency reliability of the Chinese version of the scale was very good in this study (Cronbach’s α = 0.95).

#### Disclosure of sexual orientation

Outness was measured using two questions adapted from a previous study [4]. The first question concerned the extent to which the respondents had disclosed their sexual orientation to others, with three optional categories: *never had disclosed*, *partially disclosed*, and *fully disclosed*. The responses were coded dichotomously by occurrence. The second question pertained to the extent to which the respondents had disclosed their sexual orientation to their parents. The respondents used a 5-point Likert scale ranging from 1 (*strongly disagree*) to 5 (*strongly agree*); their responses were dichotomized to reflect their disagreement/agreement with disclosure to their parents.

#### Sexual compulsivity

Sexual compulsivity was measured using the 10-item Sexual Compulsivity Scale [29] that assesses compulsive urges to perform specific sexual acts. Responses are made using a 4-point Likert scale ranging from 1 (*strongly disagree*) to 4 (*strongly agree*). Scores range from 10 to 40, with higher scores indicating a higher degree of compulsivity. The Chinese version of the scale was used in a previous study of 436 self-identified gay and bisexual men in Southwest China (Cronbach’s α = 0.91) [30].

#### Sexual orientation identity, sexual attraction, and sexual partners

Sexual identity, sexual attraction, and sexual partners were assessed using questions that were adapted from a previous online survey by Vrangalova and Savin-Williams [31]. Sexual identity was assessed by asking participants to identify their sexual orientation. They answered using one of five response options: gay, bisexual, heterosexual, questioning/uncertain, and other. Participants who self-identified their sexual orientation as heterosexual or questioning/uncertain were excluded in this study.

Sexual attraction, which included same-sex and other-sex attraction, was assessed using two separate questions: “How sexually attracted are you to women?” and “How sexually attracted are you to men?” Participants rated their attraction on a scale of 1 (*not at all*) to 5 (*very much*) [31].

Sexual partners was assessed using two separate questions: the total number of (1) male and (2) female partners with whom the respondent had sexual experiences during the previous 6 months, including vaginal sex and anal sex. The response was coded as having multiple male sex partners if the number of partners was greater than one, and it was coded as having female sex partners if the number was greater than 0.

#### Transactional sex

Transactional sex was measured using a single question: “During the previous 6 months, did you trade sex to get money?” The four response options were: *never*, *rarely*, *sometimes*, and *always*. The responses were dichotomized to reflect at least one episode of recent transactional sex.

#### Condom use

Condom use was measured using two separate questions about the frequency of condom use with male and female sex partners. There were six response options: *never*, *seldom*, *often*, *most of the time*, *every time*, and *not applicable*. The response was coded as having condomless sex if never, seldom, often, or most of the time was selected. Similar questions and recoding procedures have been used in published studies [32].

#### Substance use

To assess binge drinking, participants answered a question that was translated from a previous study [33]: “During the previous 6 months, how often [have you] had five or more drinks of alcohol in 2 hours at least once?” To assess the use of methamphetamines and Rush poppers, participants answered two separate questions: “During the previous 6 months, how often have you used methamphetamines?” and “During the previous 6 months, how often have you used Rush poppers?” Ten frequency categories were used as response options for each type of substance use. Each response was dichotomized to reflect at least one episode of recent substance use.

### Statistical analysis

The data were analyzed using IBM’s SPSS, version 17, Mplus, version 6.12, and AMOS software, version 17. Cronbach’s α was used to assess the internal consistency reliability of the instruments. To examine cultural differences, confirmatory factor analysis was performed on the Internalized Homophobia Scale. Descriptive statistics were calculated. Latent profile analysis, which is a superior statistical technique compared to the traditional method of cluster analysis and has a variety of fit indices, was used to determine the number of homogenous groups or levels based on data from the Internalized Homophobia Scale [34, 35]. Spearman’s rho correlations were computed to examine the relationships between internalized homophobia and other factors. Sexual compulsivity and psychological distress responses were later dichotomized by a median split for use in logistic regression. Univariate logistic regression was performed. Separate multivariate logistic regression analyses using block entry were then used to examine the independent association between internalized homophobia and the factors, controlling for sexual identity, current relationship status, and monthly salary.

## Results

Table 1 presents the results of the confirmatory factor analysis that was performed on the Chinese version of the Internalized Homophobia Scale. The scale’s goodness-of-fit indices were: χ^*2*^ */df* = 3.51, CFI = 0.96, TLI = 0.95, SRMR = 0.05, RMSEA = 0.08.

**Table 1:** Fit statistics for confirmatory factor model

Scale	Number	χ^*2*^ */df*	CFI	TLI	RMSEA	SRMR
Internalized Homophobia	435	3.51	.96	.95	.08	.05

*CFI* comparative fit index, *TLI* Tucker-Lewis index, *RMSEA* root mean square error of approximation, *SRMR* standardized root mean square residual

Table 2 presents respondents’ levels of internalized homophobia as measured by the Internalized Homophobia Scale. The number of bisexual respondents was small, so we combined them with the gay sample in our analyses. The mean score for internalized homophobia was 24.4 (*SD* = 7.5). Approximately 20% of the respondents agreed or strongly agreed that they had considered avoiding personal/social involvement with other gay/bisexual men, had tried to stop being attracted to men, felt alienated because of their gay/bisexual status, felt being gay/bisexual was a shortcoming, and had tried to become more sexually attracted to women. Approximately 40% of them reported that they would accept the chance to be completely heterosexual if possible, 37% of them wished they were not gay/bisexual, 31% of them wished that they could develop more erotic feelings about women, and 14% of them were willing to change their sexual orientation.

**Table 2:** Item responses of the Internalized Homophobia Scale (*N* = 435)

Items	*N*(%)			*M*	*SD*
	Disagree/Strongly disagree	Some unsure	Agree/Strongly agree		
I often feel it best to avoid personal or social involvement with other gay/bisexual Men.	158(36.3)	174(40.0)	103(23.7)	2.82	1.08
I have tried to stop being attracted to men in general.	197(45.3)	160(36.8)	78(17.9)	2.65	0.99
If someone offered me the chance to be completely heterosexual, I would accept the chance.	146(33.6)	116(26.7)	173(39.8)	3.11	1.32
I wish I weren’t gay/bisexual.	140(32.2)	134(30.8)	161(37.0)	3.07	1.26
I feel alienated from myself because of being gay/bisexual.	194(44.6)	161(37.0)	80(18.4)	2.60	1.11
I wish that I could develop more erotic feelings about women.	162(37.2)	138(31.7)	135(31.0)	2.86	1.21
I feel that being gay/bisexual is a personal shortcoming for me.	262(60.2)	88(20.2)	85(19.5)	2.37	1.20
I would like to get professional help in order to change my sexual orientation from gay/bisexual to straight.	278(63.9)	98(22.5)	59(13.6)	2.25	1.14
I have tried to become more sexually attracted to women.	183(42.1)	152(34.9)	100(23.0)	2.67	1.15

Table 3 presents the results of the latent profile analysis. We examined the plausibility of 2-, 3-, 4-, and 5-class solutions and determined the best model fit. The likelihood ratio tests were not significant for the 3- and 5-class solutions (both *ps* > 0.05), which indicated they were not suitable. The 2-class solution was better than the 4-class solution according to a larger entropy value, indicating a more accurate classification [34]. Akaike Information Criterion and the Bayesian Information Criterion both showed a moderate drop from the 2 to 4-class solutions. Thus, the 2-class solution was used to create two levels: the lower levels (*N* = 219, mean score = 18.4, *SD* = 4.2) and higher levels (*N* = 216, mean score = 30.5, *SD* = 4.5) of internalized homophobia.

**Table 3:** Latent profile analysis: model fit indexes for the 2-, 3-, 4-, and 5-class solution for internalized homophobia for gay and bisexual men (*N* = 435)

	AIC	BIC	Entropy	LRT	*P* value
2-class	11173.94	11199.20	0.86	1112.65	<0.001
3-class	10838.44	10872.71	0.84	349.75	0.70
4-class	10693.09	10736.38	0.84	162.67	0.01
5-class	10624.28	10676.59	0.84	87.38	0.30

*AIC* Akaike information criterion, *ssaBIC* sample size adjusted bayesian information criterion, *LRT* Lo-Mendell-Rubins adjusted likelihood ratio test

Table 4 presents the characteristics of the study’s sample. The majority of respondents had paid employment (92%). Most of them were single (64%) and had at least a college degree (70%). Approximately one-half of the respondents earned less than ¥2,000/$323 per month (46%). Sixty-three percent were aged 18–24 years and 37% were older than 24 years of age.

**Table 4:** Characteristics in high and low levels of internalized homophobia for gay and bisexual men (*N* = 435)

	Gay *N*(*%*)		Bisexual *N*(*%*)	
	Low IH (*N* = 190)	High IH (*N* = 136)	Low IH (*N* = 29)	High IH (*N* = 80)
Socio-demographics				
Age				
18–24 years old	119(62.6)	84(61.8)	21(72.4)	52(65.0)
≥ 25 years old	71(37.4)	52(38.2)	8(27.6)	21(35.0)
Highest education				
< College	54(28.4)	39(28.7)	10(34.5)	26(32.5)
College or more	136(71.6)	97(71.3)	19(65.5)	54(67.5)
Occupation				
Earning a job	174(91.6)	122(89.7)	27(93.1)	75(93.8)
Not earning a job	16(8.4)	14(10.3)	2(6.9)	5(6.2)
Monthly salary				
< ¥2,000/$323	93(48.9)	56(41.2)	16(55.2)	36(45.0)
> ¥2,000/$323	97(51.1)	80(58.8)	13(44.8)	44(55.0)
Relationship status				
Woman	6(3.2)	12(8.8)	6(20.7)	20(25.0)
Man	68(35.8)	34(25.0)	4(13.8)	8(10.0)
Single	116(61.1)	90(66.2)	19(65.5)	52(65.0)
Psychological distress				
Total K6 score (*Mean, SD*)	13.3(5.3)	15.6(5.8)	12.7(5.1)	13.7(5.2)
Disclosure status				
To other people (Yes)	119(62.6)	40(29.4)	11(37.9)	16(20.0)
To parents (Yes)	54(28.4)	14(10.3)	6(20.7)	12(15.0)
Sexual compulsivity (*Mean, SD*)	20.8(6.0)	24.2(6.0)	21.5(6.4)	22.3(6.5)
Sexual attraction (*Mean, SD*)				
To male	4.7(0.6)	4.5(0.6)	4.3(0.6)	4.1(0.7)
To female	1.8(0.9)	2.2(0.9)	3.1(0.6)	3.3(0.7)
Transactional sex in past 6 months				
No	183(96.3)	124(91.2)	28(96.6)	70(87.5)
Yes	7(3.7)	12(8.8)	1(3.4)	10(12.5)
Had > 1 male sex partners in past 6 months				
No	83(43.7)	48(35.3)	11(37.9)	34(42.5)
Yes	107(56.3)	88(64.7)	18(62.1)	46(57.5)
Had > 0 female sex partners in past 6 months				
No	182(95.8)	121(89.0)	19(65.5)	45(56.2)
Yes	8(4.2)	15(11.0)	10(34.5)	35(43.8)
Condomless sex				
Male (Yes)	103(54.2)	75(55.1)	16(55.2)	44(55.0)
female (Yes)	15(7.9)	22(16.2)	6(20.7)	29(36.2)
Substance use				
Alcohol (Yes)	88(46.3)	63(46.3)	20(69.0)	34(42.5)
Methamphetamine (Yes)	12(6.3)	12(8.8)	2(6.9)	4(5.0)
Rush proper (Yes)	49(25.8)	40(29.4)	7(24.1)	22(27.5)

*IH* internalized homophobia

Approximately 75% of the respondents were self-identified as gay, and 25% were bisexual. Forty-three percent disclosed their sexual orientation to others, and 20% possibly disclosed their sexual orientation to their parents. The majority of them had not experienced transactional sex during the previous 6 months (93%). Sixty percent reported having more than one male sex partner during the previous 6 months and 16% reported having female sex partners during the previous 6 months; 55% reported inconsistent condom-use during sex with males and 17% reported inconsistent condom-use during sex with females. In the previous 6 months, 47%, 7%, and 27% reported binge drinking, methamphetamine use, and Rush poppers use, respectively. The mean scores for psychological distress, sexual compulsivity, male sexual attraction, and female sexual attraction were 14.0 (*SD* = 5.5), 22.1 (*SD* = 6.3), 4.5 (*SD* = 0.6), and 2.3 (*SD* = 1.0), respectively.

Correlations showed that internalized homophobia was significantly and negatively correlated with male sexual attraction (*r* = −.25, *p* < 0.001), disclosure of their sexual orientation to others (*r* = −.36, *p* < 0.001) or to their parents (*r* = −.18, *p* < 0.001), and positively correlated with psychological distress (*r* = .14, *p* < 0.01), female sexual attraction (*r* = .34, *p* < 0.001), sexual compulsivity (*r* = .23, *p* < 0.001), transactional sex (*r* = .12, *p* < 0.05), female sex partners (*r* = .24, *p* < 0.001), condomless sex with females (*r* = .18, *p* < 0.001), and sexual orientation (*r* = .33, *p* < 0.001). However, there were no significant correlations between internalized homophobia, substance use, the number of male sex partners, and condomless sex with males.

Tables 5 and 6 present the results of the univariate and multivariate logistic regression analyses of the different levels of homophobia by study variables, respectively. In the univariate analysis, respondents who had higher levels of internalized homophobia had 1.45 higher odds of earning more than ¥2,000/$323 and 2.54 higher odds of reporting a current relationship with a woman, but 0.56 lower odds of reporting a current relationship with a man. In the multivariate analysis, after controlling for the confounding variables, the adjusted odds ratios of the men who had higher levels of internalized homophobia were significantly more likely to report psychological distress (Wald = 6.49, AOR = 1.66, *p* < 0.01), and significantly less likely to disclose their sexual orientation to others (Wald = 30.70, AOR = 0.30, *p* < .001) or to their parents (Wald = 6.72, AOR = 0.49, *p* < 0.01), than men who reported lower levels of internalized homophobia. Furthermore, the men who reported higher levels of internalized homophobia were significantly more likely to report sexual compulsivity (Wald = 14.05, AOR = 2.12, *p* < 0.001), and having transactional sex during the previous 6 months (Wald = 5.23, AOR = 2.77, *p* < 0.05), and had significantly lower odds of female sexual attraction (Wald = 20.50, AOR = 1.76, *p* < 0.001). In addition, although not statistically significant but more away from the null, men who reported higher levels of internalized homophobia were more likely to self-identify as being bisexual (Wald = 1.73, AOR = 1.48, *p* = 0.06) and less likely to report male sexual attraction (Wald = 3.36, AOR = 0.73, *p* = 0.07). Further, they were more likely to have had female sex partners during the previous 6 months (Wald = 2.90, AOR = 1.85, *p* = 0.09) and have condomless sex with females (Wald = 3.47, AOR = 1.83, *p* = 0.06).

**Table 5:** Univariate analysis: factors associated with a high level of internalized homophobia (*N* = 435)

	Wald	OR(95% CI)	*p* value
Socio-demographics			
Age ≥ 25 years old	0.00	1.00(0.68,1.48)	NS
< College education	0.17	1.09(0.72,1.64)	NS
Earning a job	0.05	0.93(0.47,1.82)	NS
Monthly salary > ¥2000/$323	3.72	1.45(1.00,2.12)	0.05
Relationship with a woman	6.71	2.54(1.25,5.13)	0.01
Relationship with a man	6.67	0.56(0.35,0.87)	0.01
Sexual orientation			
Bisexual (Gay referent)	30.54	3.85(2.39,6.22)	0.001
Psychological distress (higher score)	8.50	1.76(1.20,2.57)	0.001
Disclosure status			
To other people (No)	47.43	4.17(2.78,6.27)	0.001
To parents (No)	15.43	2.76(1.66,4.57)	0.001
Sexual compulsivity (higher score)	14.91	2.13(1.45,3.12)	0.001
Sexual attraction			
To male	17.01	0.51(0.37,0.71)	0.001
To female	43.75	2.00(1.63,2.46)	0.001
Transactional sex in past 6 months (Yes)	6.66	2.99(1.30,6.88)	0.01
Had > 1 male sex partners in past 6 months (Yes)	1.11	1.23(0.84,1.80)	NS
Had > 0 female sex partners in past 6 months (Yes)	15.13	3.09(1.75,5.45)	0.001
Condomless sex			
Male (Yes)	0.12	1.07(0. 73,1.56)	NS
female (Yes)	12.86	2.70(1.57,4.64)	0.001
Substance use			
Alcohol (Yes)	1.24	0.81(0.55,1.18)	NS
Methamphetamine (Yes)	0.17	1.17(0.56,2.46)	NS
Rush proper (Yes)	0.27	1.12(0.73,1.71)	NS

*OR* odds ratio, *CI* confidence interval, *NS* no significance

**Table 6:** Multivariate analysis: factors associated with internalized homophobia (*N* = 435)

	Wald	AOR	Wald	AOR	Wald	AOR	Wald	AOR	Wald	AOR
Socio-demographics										
Relationship with a woman	1.40	1.61(0.73,3.52)	**4.62**	**2.22(1.07,4.60)**	2.15	1.78(0.82,3.84)	0.25	1.24(0.53,2.89)	**3.90**	**2.10(1.01,4.36)**
Relationship with a man	**5.06**	**0.57(0.35,0.93)**	**6.21**	**0.56(0.36,0.88)**	**4.75**	**0.59(0.36,0.95)**	**6.51**	**0.55(0.34,0.87)**	**7.90**	**0.52(0.33,0.82)**
Monthly salary > ¥2000/$323	**3.61**	**1.52(0.99,2.33)**	1.19	1.25(0.84,1.86)	1.25	1.27(0.83,1.94)	0.31	1.12(0.75,1.69)	1.28	1.26(0.84,1.89)
Sexual orientation	1.73	1.48(0.82,2.67)								
Attraction to male	3.36	0.73(0.52,1.02)								
Attraction to female	**20.5**	**1.76(1.38,2.25)**								
Psychological distress (higher score)			**6.49**	**1.66(1.12,2.44)**						
Disclosure status to all people (No)					**30.7**	**0.30(0.20,0.46)**				
Disclosure status to parents (No)					**6.72**	**0.49(0.28,0.84)**				
Transactional sex in past 6 months (Yes)							**5.23**	**2.77(1.16,6.65)**		
Had > 1 male sex partners in past 6 months (Yes)							0.27	1.12(0.74,1.70)		
Had > 0 female sex partners in past 6 months (Yes)							2.90	1.85(0.91,3.77)		
Condomless male sex (Yes)							0.00	0.99(0.66,1.50)		
Condomless female sex (Yes)							3.47	1.83(0.97,3.44)		
Sexual compulsivity (higher score)									**14.1**	**2.12(1.43,3.13)**

*AOR* adjusted odds ratio

Bold values are statistically significant

For separate multivariate analysis, we controlled for current relationship status, and monthly salary

## Discussion

This descriptive study first examined the suitability of the Internalized Homophobia Scale using confirmatory factor analysis in a sample of Chinese gay and bisexual men. The Chinese version of the instrument was found to have acceptable psychometric properties. More importantly, the study found a relatively high level of internalized homophobia among the Chinese gay and bisexual men, and a positive association between higher levels of internalized homophobia with psychological distress, sexual attraction to females, transactional sex during the past 6 months, having female sex partners and condomless sex with females during the past 6 months, and sexual compulsivity. Higher levels of internalized homophobia were negatively associated with respondents’ disclosure of sexual orientation to other people and their parents, sexual attraction to males, and self-identification as bisexual.

The high levels of internalized homophobia among the gay and bisexual men in this study can be explained by the reality that Chinese social attitudes toward male sexual minorities have been negative [16, 36]. Compared to male sexual minorities outside China [18], those in China, such as gay men, are sometimes considered to be synonymous with HIV/AIDS [15]. Even in Shanghai, a highly cosmopolitan city, MSM reported feeling marginalized and experienced many forms of social discrimination and negative attitudes from multiple groups of people, such as their families, colleagues, and employers [17]. The values of marriage and family responsibilities, which are emphasized in traditional Chinese values, contribute to the development of internalized homophobia among gay and bisexual individuals. The results of this study indicated that more than 30% of the respondents wished they were not gay/bisexual men, would like to be heterosexual, and wished they could develop more erotic feelings about women. As reported in previous studies, individuals with internalized homophobia who were ashamed of their sexual orientation were less likely to have HIV testing and less likely to be exposed to intervention messages or healthcare services targeted to gay/bisexual men [6, 18].

These findings are consistent with our hypothesis that internalized homophobia is positively associated with psychological distress. The hypothesis is also supported by previous studies, which reported that individuals experiencing higher levels of internalized homophobia were more likely to have high levels of psychological distress [19, 37]. According to Meyer’s minority stress theory, gay and bisexual individuals experience conflicting sexual values and a social environment relevant to their same-sex attraction. Once a person self-identifies as being gay, the recognition of their “gay” status has psychologically injurious effects due to social homophobia. Thus, it begins to threaten their psychological well-being [27] and may explain why gay and bisexual individuals are more likely to have suicidal ideation and attempts [38]. In addition, these findings offer an Eastern cultural perspective supporting Southwest Chinese gay and bisexual men and the associations between their internalized homophobia and psychological distress.

After adjusting for the significant confounding variables, internalized homophobia was significantly associated with respondents’ reports of transactional sex during the previous 6 months; and it had, to some extent, a weak association with condomless sex with females and having female sex partners during the previous 6 months. Previous studies have reported a high proportion of transactional sex among MSM in Africa [6, 39], but no associations between internalized homophobia and transactional sex [39]. Although this finding indicates there is a low occurrence of transactional sex among male sexual minorities in China, individuals were more likely to report higher levels of internalized homophobia. Thus, the association between internalized homophobia and transactional sex is inconclusive and might be influenced by race and culture. Unlike the finding of a previous study [40], the present findings suggest that individuals with higher levels of internalized homophobia were more likely to have sex with females, although this finding was not statistically significant but more away from the null. The study also revealed that men with higher levels of internalized homophobia were to some extent more likely to engage in unprotected sex with females. Given the dramatic increase in HIV transmission among Chinese MSM [14, 18], these findings suggest the potential for bridging the transmission between Chinese gay/bisexual men, especially those with higher levels of internalized homophobia, and the general population through female sex partners. However, no relationship was found between internalized homophobia and substance use in this sample. Similar to a previous review, mixed results have been reported in studies of these relationships [41]. Cultural and social consciousness might determine these associations.

Higher levels of internalized homophobia were significantly associated with a lower level of disclosure to others and parents. It is possible that the men with higher levels of internalized homophobia were more likely to experience sexual minority stress, thereby engaging in behaviors to protect themselves from harm [5], such as rejection by parents, relatives, and friends, and threats to employment. They might have also developed coping behaviors to comfort themselves. This information may also be applied to helping members of this population solve problems related to minority stress and to improve the mental health of individuals who cope with concealing their sexual orientation. We also found that respondents with higher levels of internalized homophobia were significantly less likely to report male sexual attraction and more likely to report female sexual attraction. Previous studies showed that sexual orientation was generally consistent with sexual attraction [31]. This finding supports the finding of Vu and Sheehy’s study that MSM who did not identify themselves as gay were more likely to report internalized homophobia [6]. In addition, we found a relationship between internalized homophobia and sexual compulsivity. These findings are consistent with those of past studies that reported a negative association between internalized homophobia and gay/bisexual individuals’ mental health, which includes addictive and psychosomatic symptoms [20, 26].

The study has several limitations. First, the data are cross-sectional; thus, causal relationships cannot be inferred. Additionally, some of the associations were weak, such as the association between internalized homophobia and transactional sex during the previous 6 months. Second, participants were recruited via the Internet, which has the disadvantage of self-selection and might have resulted in the underrepresentation of certain populations, e.g., older respondents. Third, certain topics requiring self-report, such as amphetamine use and transactional sex are sensitive in the Chinese culture, which might have biased the responses due to social desirability [42]. Fourth, measure of the choice of disclosure of sexual orientation to others and parents was not fully conceptualized compared with previous studies [3, 5]. Further studies using an adequate definition and measure of outness are needed to understand the experiences of Chinese male sexual minorities.

## Conclusion

This study has important implications because it is the first to examine the prevalence and correlates of internalized homophobia among Chinese gay and bisexual men. It also provides a much-needed cross-cultural perspective, as it was conducted in the context of the Chinese culture. High levels of internalized homophobia were found among Chinese male sexual minorities, and internalized homophobia was positively associated with psychological distress, transactional sex during the previous 6 months, and the concealment of sexual identity to others and parents. The Chinese government’s involvement, such as MSM community-based advocacy efforts as well as government-enforced policies in the prevention of internalizing homophobia among gay/bisexual men, is important to buffer the negative effects of a homophobic society on the mental health of this population. Decreasing their gay-related psychological distress and stress, as well as HIV transmission via male-to-female sex, should improve the public health of all.

## Additional file

Additional file 1: Questionnaire of the study. (DOCX 31 kb)

## Abbreviations

AIC: Akaike information criterion
AOR: Adjusted odds ratio
CFI: Comparative fit index
CI: Confidence interval
IH: Internalized homophobia
LRT: Lo-Mendell-Rubins adjusted likelihood ratio test
NS: No significance
RMSEA: Root mean square error of approximation
SRMR: Root mean square residual
ssaBIC: Sample size adjusted bayesian information standardized criterion
TLI: Tucker-lewis index
