# Supplementary material for: Internalized homophobia, mental health, sexual behaviors, and outness of gay/bisexual men from Southwest China
Source: Int J Equity Health. 2017 Feb 17;16:36. doi: 10.1186/s12939-017-0530-1 (PMC5320694; doi:10.1186/s12939-017-0530-1)
Supplement: Additional file 1: — Questionnaire of the study. (DOCX 31 kb) [file 12939_2017_530_MOESM1_ESM.docx]

**Survey**

**1. What is your age? (Arabic numeral)**

_____________

**2. What is your gender?**



Female



Male



Transgender

**3. What is your highest educational level?**



Primary or junior high school diploma

Senior high school diploma



University or college degree



Postgraduate or above

**4. What is your occupation status?**

Earning a job

Not earning a job

**5. What is your monthly salary?**



None



Less than ¥2,000



¥2,000-¥3,999

¥4,000-¥5,999



¥6,000-¥9,999

More than ¥9,999

**6. What is your current relationship status?**



Having a relationship with a woman

Having a relationship with a man



Single

**7. A female has sex appeal for you?**



No

A little

Some

Much



Very much

**8. A male has sex appeal for you?**



No

A little

Some

Much



Very much

**9. Have you disclosed your sexual orientation to others?**

Never

Partially



Fully

**10. Did you trade sex to get money in the past 6 months?**



Never



Rarely

Sometimes

Always

**11. What is your sexual orientation?**



G**ay**

Bisexual

Heterosexual

Questioning/Uncertain

Others

**12. Do you agree or disagree with the statement that it is ok to disclose your sexual orientation to your parents?**



Never

Somewhat disagree

Neither agree nor disagree

Somewhat agree

Strongly agree

**13. What is the total number of male partners who have had vaginal sex with you in the past 6 months?**



0

1

2

3

4

5

6-7

8-10

11-20

Over 20

**14. What is the total number of male partners who have had anal sex with you in the past 6 months?**



0

1

2

3

4

5

6-7

8-10

11-20

Over 20

**15. How many times have you had 5 or more drinks of alcohol in two hours in the past 6 months?**



Never

Once every six months

Once every two or three months

Once a month

Once every two weeks

Once a week

Once every two or three days

Once every day

Several times every day

Unwilling to answer

**16. How many times have you used methamphetamines in the past 6 months?**



Never

Once every six months

Once every two or three months

Once a month

Once every two weeks

Once a week

Once every two or three days

Once every day

Several times every day

Unwilling to answer

**17. How many times have you used Rush poppers in the past 6 months?**



Never

Once every six months

Once every two or three months

Once a month

Once every two weeks

Once a week

Once every two or three days

Once every day

Several times every day

Unwilling to answer

**18. According to your personal situation, choose the most suitable option please. [Internalized Homophobia Scale]**

**A. I often feel it best to avoid personal or social involvement with other gay/bisexual**

**Men.**



Strongly disagree

Somewhat disagree

Neither agree nor disagree

Somewhat agree

Strongly agree

**B. I have tried to stop being attracted to men in general.**



Strongly disagree

Somewhat disagree

Neither agree nor disagree

Somewhat agree

Strongly agree

**C. If someone offered me the chance to be completely heterosexual, I would accept the chance.**



Strongly disagree

Somewhat disagree

Neither agree nor disagree

Somewhat agree

Strongly agree

**D. I wish I weren’t gay/bisexual.**



Strongly disagree

Somewhat disagree

Neither agree nor disagree

Somewhat agree

Strongly agree

**E. I feel alienated from myself because of being gay/bisexual.**



Strongly disagree

Somewhat disagree

Neither agree nor disagree

Somewhat agree

Strongly agree

**F. I wish that I could develop more erotic feelings about women.**



Strongly disagree

Somewhat disagree

Neither agree nor disagree

Somewhat agree

Strongly agree

**G. I feel that being gay/bisexual is a personal shortcoming for me.**



Strongly disagree

Somewhat disagree

Neither agree nor disagree

Somewhat agree

Strongly agree

**H. I would like to get professional help in order to change my sexual orientation from**

**gay/bisexual to straight.**



Strongly disagree

Somewhat disagree

Neither agree nor disagree

Somewhat agree

Strongly agree

**I. I have tried to become more sexually attracted to women.**



Strongly disagree

Somewhat disagree

Neither agree nor disagree

Somewhat agree

Strongly agree

**19. According to your personal situation, choose the most suitable option please. [Sexual Compulsivity Scale]**

**A. My sexual appetite has gotten in the way of my relationships.**



Strongly disagree

Somewhat disagree

Somewhat agree

Strongly agree

**B. My sexual thoughts and behaviors are causing problems in my life.**



Strongly disagree

Somewhat disagree

Somewhat agree

Strongly agree

**C. My desires to have sex have disrupted my daily life.**



Strongly disagree

Somewhat disagree

Somewhat agree

Strongly agree

**D. I sometimes fails to meet my commitments and responsibilities because of my sexual behaviors.**



Strongly disagree

Somewhat disagree

Somewhat agree

Strongly agree

**E. I sometimes get so horny I could lose control.**



Strongly disagree

Somewhat disagree

Somewhat agree

Strongly agree

**F. I find myself thinking about sex while at work.**



Strongly disagree

Somewhat disagree

Somewhat agree

Strongly agree

**G. I feel that my sexual thoughts and feelings are stronger than I am.**



Strongly disagree

Somewhat disagree

Somewhat agree

Strongly agree

**H. I have to struggle to control my sexual thoughts and behavior.**



Strongly disagree

Somewhat disagree

Somewhat agree

Strongly agree

**I. I think about sex more than I could like to.**



Strongly disagree

Somewhat disagree

Somewhat agree

Strongly agree

**J. It has been difficult for me to find sex partners who desire having sex as much as I want to.**



Strongly disagree

Somewhat disagree

Somewhat agree

Strongly agree

**20. How frequently did you use condoms with female sex partners?**

| Never  Seldom  Often  Most of the time  Every time  Not applicable |  |  |
| --- | --- | --- |

**21. How frequently did you use condoms with male sex partners?**

| Never  Seldom  Often  Most of the time  Every time  Not applicable |  |  |
| --- | --- | --- |

**22. During the last 30 days, about how often did** _______**? [Kessler Psychological Distress Scale]**

**A. you feel so depressed that nothing could cheer you up?**

| None of the time  A little of the time  Some of the time  Most of the time  All of the time  **B. you feel hopeless?**  None of the time  A little of the time  Some of the time  Most of the time  All of the time  **C. you feel restless or fidgety?**  None of the time  A little of the time  Some of the time  Most of the time  All of the time  **D. you feel that everything was an effort?**  None of the time  A little of the time  Some of the time  Most of the time  All of the time  **E. you feel worthless?**  None of the time  A little of the time  Some of the time  Most of the time  All of the time  **F. you feel nervous?**  None of the time  A little of the time  Some of the time  Most of the time  All of the time |
| --- |
